# Evaluation of the Quality of Four Root Canal Obturation Techniques Using Micro-Computed Tomography

**Published:** 2013-08-01

**Authors:** Mandana Naseri, Ali Kangarlou, Atefeh Khavid, Mostafa Goodini

**Affiliations:** a Iranian Center for Endodontic Research, Research Institute of Dental Sciences, Shahid Beheshti University of Medical Sciences, Tehran, Iran; b Department of Endodontics, Dental School, Shahid Beheshti University of Medical Sciences, Tehran, Iran; c Students Research Committee, Shahid Beheshti University of Medical Sciences, Tehran, Iran

**Keywords:** Computed Tomography, Micro-Computed Tomography, Root Canal Filling Materials, Root Canal Obturation

## Abstract

**Introduction:**

One of the key factors in successful endodontic therapy is to adequately fill the root canals. The aim of this in vitro study was to compare the quality of four different root canal obturation techniques: cold lateral condensation (CLC), warm vertical condensation (WVC), Obtura II (OII) and Gutta Flow (GF) by using micro-computed tomography (micro CT).

**Materials and Methods:**

A total of 20 extracted maxillary first molars prepared with ProTaper files, were randomly divided into four groups. Micro CT was used to measure the internal volume of root canals. Following application of AH26 sealer to canal obturation, new micro-CT images were taken and the volume percentage (VP) of voids, gutta-percha and sealer at different levels were calculated with CT software. Data was statistically analyzed using Kruskal-Wallis and Mann-Whitney U tests.

**Results:**

The highest percentage of filling material was observed in GF group followed by OII with no statistically significant difference (P>0.05). These two groups had a significantly more acceptable filling than WVC and CLC groups (P<0.05). Voids were detected in all samples. There was a significant difference between the highest and the lowest percentage of voids in CLC (19.6%) and GF groups (6.7%), respectively. In the apical third, CLC and OII showed the highest and the lowest percentage of voids (5.5% and 2.6%) and the lowest and highest percentage of gutta-percha (76.52% and 94.26%), respectively. These differences were statistically significant (P<0.05).

**Conclusion:**

None of the root canal filled teeth were gap-free. GF and CLC techniques showed the highest and lowest VP of obturation materials, respectively.

## Introduction

LOng term success in endodontic treatment is due to three-dimensional obturation of root canal(s) in order to prevent ingress of bacteria and their toxins into the periapical tissues [1, 2]. Root canal filling with no voids and obturation to within 2 mm of the apex are among the factors affecting the efficacy of primary root canal treatment based on a meta-analysis [3].

Suitable physical properties of Gutta-percha (GP) as the most commonroot canal obturation material, allow it to apply in several obturation techniques [4]. Although cold lateral condensation is the most commonly used technique, but voids, spreader tracts, incomplete fusion of GP cones, and lack of surface adaptation are among the reported drawbacks [5]. Thermoplasticized injectable techniques were introduced to improve the homogeneity and surface adaptation of GP. Overfilling occurred in 75% of cases with vertical condensation of thermoplasticized GP [6]. In order to overcome the flaws *i.e.* apical extrusion and shrinkage in thermoplasticized condensation, cold free-flow obturation technique was introduced. According to the manufacturer, Gutta Flow has excellent flow properties because its viscosity diminishes under shear stresses [7]. This material flows into lateral canals and since no heat is required for its placement so no shrinkage is believed to occur [7].

Various experimental methods have been used to assess the quality of root fillings, such as: radioisotope, dye penetration, fluid filtration, bacterial leakage, microscopic analysis, clearing techniques and micro-computed tomography (micro-CT) [8-15]. In endodontics, micro-CT has been used for evaluation of root canal anatomy and morphology following instrumentation [16, 17]. This method has the advantages of highly accuracy and being nondestructive [8]. There are only a few studies available evaluating the obturation quality by micro-CT [14, 18, 19].

The aim of this *in vitro* study was to compare the quality of four different root canal obturation techniques: cold lateral condensation, warm vertical condensation, Obtura II and Gutta Flow by using micro-computed tomography.

## Material and Methods

### Tooth specimens

Twenty extracted mature maxillary first molars with three distinct roots and no root caries, restoration, apical resorption or previous endodontic treatment were selected. In order to standardization of samples, all of them had a curvature less than 15 degrees, as determined by the Schneider’s method [20]. The teeth were immersed in 5.25% sodium hypochlorite for one day, then stored in normal saline solution during the study period.

### Tooth preparation

After preparing a standard access cavity in each tooth, a #10 K-file was introduced into the canals until the tip was just visible at the apical foramens. The working length (WL) was determined 0.5 mm short of this measurement. After hand file using and establishing a glide path, ProTaper files (Dentsply Tulsa Dental, Tulsa, OK, USA) were used according to the manufacturer’s protocol to clean and shape the canals up to F3, using 1 mL of 2.5% sodium hypochlorite as an irrigant between each two files. After complete instrumentation, all specimens received a final flush of 1 mL 17% EDTA followed by 5 mL 2.5% NaOCl for smear layer removal [9].

Micro-CT scanner was used to scan the teeth. After adjusting appropriate parameters for scanning, each tooth was positioned on the specimen stage and scanned by a high-resolution micro-CT scanner (SkyScan-1072, Kontich, Belgium). Each image had a resolution of 1024×1024 pixels, a voxel size of 19.5×19.5×39.5 µm, rotational step of 0.90 degree, rotational angle of 180 degrees, and a 3-second exposure time. By using the NRecon software (Skyscan, Kontich, Belgium), the images obtained by the scanner were reconstructed to show 2-dimensional slices of the roots. The CTVol (Skyscan, Aartselaar, Belgium) software was used for the 3-dimensional volumetric visualization, analysis, and volume of the root canal measurement. The area of prepared root canal in each slice was measured from the orifice of canals to the apical constriction. The volume of root canal in each slice was calculated by multiplying the root canal area by the slice thickness (0.5 mm). The root length was divided into three equal coronal, middle and apical thirds and the volume of each segment was calculated separately.

### Filling of the root canal

AH26 sealer (Dentsply Maillefer, Ballaigues, Switzerland) was placed inside the canals using Lentulo-spiral by one operator and the teeth were randomly divided into four groups of 5 samples.

*Group I* [Cold lateral condensation (CLC)]: Lateral condensation was done using standardized GP as the master cones, finger spreaders (B and C) (Maillefer, Ballaigues, Switzerland) and medium-fine accessory cones (Dentsply Maillefer, Ballaigues, Switzerland). The excess GP at the orifices was removed by a heated instrument and final compaction was done by a cold plugger (Dentsply Mailleffer, Paris, France)

*Group II* [Warm vertical condensation (WVC)]: A fine-medium or medium-sized GP cone was selected as the master cone. Gently inserting into the canals, the GP point was fitted within 1 mm of the WL. Coronal portion of GP was cut and apically condensed. After packing gutta-percha to within 4 mm of the apex, 3-4 mm long segments of gutta-percha were backpacked until the canals were completely obturated.

*Group III* [Obtura II (OII)]: According to the manufacturer’s instructions, a 20 gauge Obtura (Obtura Spartan, Fenton, MI, USA) cannula tip was selected and inserted into the canal 3–5 mm short of the WL. The temperature was set at 200˚C, the trigger was pressed allowing the molten GP to flow and the tip was withdrawn slowly out of the canal. The apical segment was compacted using appropriate Obtura pluggers. Backfilling was achieved by the application of thermoplasticized GP in 4-5 increments, followed by uniform compaction with pluggers.

*Group IV* [Gutta Flow (GF)]: Following the manufacturer’s instructions, the GF plastic insertion tip was placed into the canal to a depth at which the tip no longer advanced. The GF filling depth starting point was established 3 mm short of this length. The GF capsule was activated and the plastic tip was attached to the capsule. Inserting the tip into the canal to the filling depth, the material was dispensed. A standardized GP master cone was coated with GF and inserted to the WL. The cone was gently pulled upward 2 to 3 mm, twisted twice and reseated to the WL. The canal was backfilled with GF by placing the plastic insertion tip next to the master point to a depth at which the tip was neither forced nor bound the canal wall.

After canal obturating and sealing the access cavities by Coltosol, (Ariadent, Tehran, Iran) all the teeth were stored at 37˚C with 95% humidity for about 72 hours to complete setting of sealers. Then a second micro-CT scan was performed to determine the volume of GP and sealer defined as volume percentage (VP) in the coronal, middle and apical thirds of each canal. 

Specialized CT software was used to measure the VP of voids in the obturated root canals.

### Statistical analysis

Statistical analysis was performed with nonparametric tests (Kruskal-Wallis and Mann-Whitney U tests). The level of significance was set at *P*<0.05.

## Results

**Figure 1.**
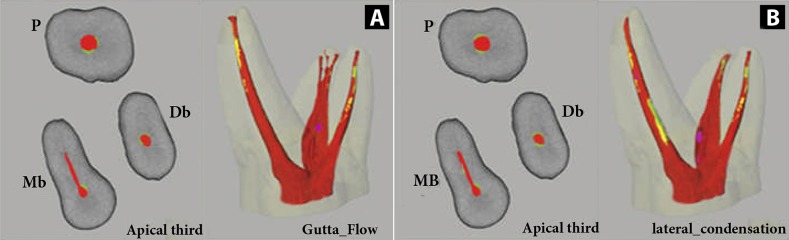
Three-dimensional (Right) and 2D (Left) reconstructed models of the obturated root canals by Gutta Flow (A) and cold lateral condensation (B); separating GP (red), sealer (yellow and green) and voids (violet).

All volume percentage (VP) values at different levels of root canals are summarized in Table 1. The highest percentage of filling material in the apical third and the whole length of the root canal was observed in the GF and OII groups with no statistically significant difference between them (*P*>0.05); however, in comparison, the percentage of filling material in these two groups was significantly higher than that of CLC and WVC groups (*P*<0.05) (Figure 1).

**Table 1.** VP values for each group at different levels of root canals [mean (SD)].

Groups	Overall	Coronal third	Middle third	Apical third
**Cold Lateral Condensation**	80.4 (1.6)	80.7 (3.6)	81.5 (3.1)	83 (3.60)
**Warm Vertical Condensation**	84.8 (6.0)	92.9 (8.5)	95.6 (6.1)	89.3 (9.3)
**Obtura II**	92.7 (2.4)	92.5 (3.4)	95.5 (4.7)	97.4 (5.4)
**Gutta Flow**	93.3 (3.6)	92.1 (5.7)	94.7 (6.0)	96.2 (6.2)

VP values for GP and sealer are summarized in Table 2. Overall, OII and CLC techniques showed the highest (84.38%) and the lowest (62.74%) percentage of GP, respectively. However, no statistically significant difference was detected between OII and GF groups in this respect (*P*>0.05). Although GF group had the highest overall VP (93.3±3.6), when comparing the ratio of VP of GP to the overall obturated volume, OII group showed the greatest value (84.38%). However, the difference between GF (82.59%) and OII in this respect was not statistically significant. In the apical third, CLC technique showed the lowest percentage of GP (76.52%) and no statistically significant difference was detected among the remaining three groups (*P*>0.05).

**Table 2.** Volume Percentages for Gutta-percha and sealer (µm3)

Groups	Volume Percentage [mean (SD)]		Gutta-Percha (%)		Sealer (%)	
	Overall	Apical third	Overall	Apical third	Overall	Apical third
**Cold Lateral Condensation**	80.4 (1.6)	83 (3.6)	62.47	76.52	17.93	6.84
**Warm Vertical Condensation**	84.8 (6.0)	89.3 (9.3)	73.02	85.84	11.78	3.46
**Obtura II**	92.7 (2.4)	97.4 (5.4)	84.38	94.26	8.32	3.14
**Gutta Flow**	93.3 (3.6)	96.2 (6.2)	82.59	92.92	10.71	3.28

Based on the measurements of the voids which were detected in all samples, these results were obtained: the highest VP (19.6%) was detected in CLC technique which in comparison with GF, it was significantly higher than GF with the lowest VP (6.7%). Also, in the apical third, OII and CLC had the lowest (2.6%) and the highest (5.5%) VP of voids, respectively (Table 3).

**Table 3.** Percentage of voids in each group (µm3)

Groups	Overall	Apical Third
**Cold Lateral Condensation**	19.6	5.5
**Vertical Condensation**	15.2	4.7
**Obtura II**	7.3	2.6
**Gutta Flow**	6.7	3.8

## 4. Discussion

Various experimental methods have been introduced to assess the quality of root canal fillings. Conventional methods of evaluating root fillings have some disadvantages; on sectioning the root, there could be loss of material which might mimic voids, radiographs give only two-dimensional interpretations [21], the time taken for fluid filtration [22] and clearing techniques [23] may be a concern, dye penetration studies do not correlate clinically [24] and dye extraction studies evaluate only the apical third of the tooth [25]. Bacterial microleakage studies need long periods of observation and do not allow quantification of the number of penetrating bacteria [26, 27]. Micro-CT analysis can provide high-resolution images as well as both qualitative and quantitative analysis of tooth, bone and implants. This method not only is rapid and non-invasive but also the results are reproducible and comparable with histologic studies [28]. Additionally, the segmentation of closely related objects such as different dental hard tissues, calcified tissues and root canal filling material is possible [18]. However, *in-vivo* application of micro-CT technique has various limitations. Other problems can be due to the chosen segmentation threshold values, which may affect the appearance of the objects of interest [29]. In the field of endodontic research, there are only a few studies focusing on micro-CT analysis of obturated root canals [18, 19].

Numerous *in vitro* investigations have evaluated obturation techniques by comparing different variables such as length of fill [30], defect replication [31] and GP density [32]. In the current study, we performed a volume analysis with micro-CT in which the focus was on the volume of GP and sealer. Micro-CT allows for three-dimensional volume measurements without sectioning the specimens and thus avoiding the loss of material during sectioning [33]. In addition, it can distinguish GP and sealer by different colors.

It is important to obturate the whole length of root canal. However, since the apical third is especially important, all the measurements were done separately for the apical third as well.

Based on the present study results, voids were detected in all samples. The highest overall VPof void was detected in CLC group which was significantly higher than GF with the lowest volume of voids. In the apical third, OII showed the lowest percentage of voids not significantly different from the GF group but in contrast with CLC with the highest VPof voids. The difference in this regard between the two groups was statistically significant.

In this study GF followed by OII techniques showed the highest overall VPof obturation material in comparison to CLC and WVC at all levels of the root canal. The reason may be better flowability and increased wettability of GP in the GF group compared to other groups. In addition, use of heat-softened GP resulted in a better homogenous mass with less voids and improved adaptation to the canal walls in OII technique. This is in accordance with some studies [34, 35], but in contrast with others [36], CLC group had the lowest value of overall filling volume among the experimental methods. This may be attributed to the fact that this technique does not produce a homogenous mass and may leave gaps between the GP and the dentinal walls or accessory cones. One study showed that the final filling in CLC group had the appearance of numerous GP cones tightly pressed together and joined by frictional grip and the cementing substance, while the spreader tracts can be devoid of sealer or the sealer can resorb later leading to voids [37]. Our study showed that there were voids between the accessory cones throughout the length of the canal in CLC group.

According to some studies the more thickness of sealer, the lower seal ability of the method [38, 39]. One of the advantages of using micro-CT for evaluation of obturation techniques is its ability to differentiate the VPof GP and sealer in the obturating material, hence assessing the quality of obturation technique to fill the canal by GP, not the sealer. This is especially important because of the sealers’ wash out over time [37]. The results of the present study showed that OII had the highest percentage of GP in the obturating material in both the apical third and whole length of the root canal, while CLC ranked lowest in this regard.

## 5. Conclusion

The present *in vitro *study demonstrated that none of the root canal filled teeth were gap-free; GF and CLC obturation techniques had the highest and the lowest VP of obturation materials, respectively.
